# Supplementary material for: How does the audience return to the music theater? Exploring the influence of musical theater adaptation of Chinese nursery rhymes
Source: PLoS One. 2025 May 20;20(5):e0321689. doi: 10.1371/journal.pone.0321689 (PMC12091828; doi:10.1371/journal.pone.0321689)
Supplement: Attachment — Submitted filename: Response to Reviewers.pdf [file pone.0321689.s002.pdf]

Dear Reviewers,

I hope this message finds you well. I would like to express my sincere gratitude for taking the time to review our manuscript titled "How does the Audience Return to the Music Theater? Exploring the Influence of Musical Theater Adaptation of Chinese Nursery Rhymes." Your insightful comments and constructive suggestions have been invaluable in enhancing the quality of our work. We have carefully considered each of your feedback points and have made comprehensive revisions accordingly. Below, I have outlined the specific changes made in reactions to your recommendations:

We have refined the introduction to articulate the research objectives, emphasizing the unique focus more clearly on the impact of nursery rhyme adaptations in musicals on audience interaction, affective reactions, satisfaction, and repurchase intention. This has helped to frame the study within the current market context in China, where musicals are gaining prominence. Following your suggestions, we have provided additional details on the questionnaire design, sampling strategy, and data collection procedures to ensure transparency and reproducibility. Furthermore, we have incorporated a more rigorous validation process for the measurement scales used in the study.

We have expanded the results section to include a more thorough analysis of how audience interaction and affective reactions enhance satisfaction and increase repurchase intention. Specifically, we have included additional statistical tests and graphical representations to support our findings. Building upon your suggestions, we have refined our strategic recommendations to emphasize the crucial role of audience interaction and affective reactions throughout the creative process. These recommendations now include specific actionable steps for musical producers to enhance audience engagement and satisfaction. In the conclusion, we have further elaborated on the theoretical and practical insights gained from our study, underscoring its contributions to cultural inheritance and innovation. We have also highlighted potential avenues for future research to continue advancing this field.

We believe that these revisions have significantly improved the manuscript, making it more robust, coherent, and impactful. We are confident that the revised version now addresses all the concerns raised and presents a valuable contribution to the field of musical theater studies. Once again, thank you for your invaluable feedback and dedication to improving the quality of our work. We look forward to the next steps in the publication process and hope to see our manuscript published in PLOS ONE.

Warm regards,

Jian Li

ZhanJiang Preschool Education Collage, Zhanjiang, China

## Response to Reviewers' Comments :

Reviewer #1:
1.Replace Inactive Links: Some external links cited in the article are inactive, especially in the research background and literature review sections, which affects the retrieval of literature. Please check and replace them with active links or directly cite accessible literature to ensure the rigor and traceability of the research.

We appreciate your feedback. We acknowledge the issue with inactive links and have thoroughly reviewed all external citations in the research background and literature review sections. We have replaced any inactive links with active ones or directly cited accessible literature to ensure rigor and traceability. The updated references have been verified for accuracy.

> and form a brand effect (Hanxi(2023) [17]).
>
> ~~According to Strategy Helix's "China's Performing Arts Market" study~~According to Strategy Helix's "China's Performing Arts Market" study, in China, after a significant contraction (-54.9%) in 2020, the revenue of the performing arts market has increased significantly (37.3%), reaching 5.162 billion in ~~2021[1].~~ 2021. The musical theater market has a very small scale, indicating that the number of domestic musical theater projects is relatively small, and the supply and demand sides of the market do not match, resulting in limited audience choices and skyrocketing ticket prices. Therefore, this study combines the audience's perception of the price of musicals and evaluates both affective and cognitive responses to reflect the symbiotic cycle that exists in actual musical decision-making.

Reviewer #1:
2.Supplement Research Methods: The article lacks a detailed description of the research methods, making it difficult to understand the specific implementation process and the basis for method selection. It is recommended to organize the research methods separately, covering aspects such as research design, data collection, sample selection, data processing, and analysis, and connect them with the theoretical background or hypothesis section to provide methodological support for hypothesis verification and theoretical discussion.

We appreciate your helpful review and comments. We understand the importance of a detailed description of the research methods. We have reorganized the methods section to comprehensively cover research design, data collection, sample selection, data processing, and analysis. These details are now clearly articulated and linked to the theoretical background and hypothesis section, providing robust methodological support for hypothesis verification and theoretical discussion.

### 4.2 Participant Demographics

The participants of the questionnaire were audience members who had watched performances at two theaters in Guangzhou, and a total of 326 responses were received. After excluding 15 responses that did not meet the inclusion criteria, 311 valid responses were retained for analysis. This study used a random sampling method to investigate these audiences (the results are shown in Table 2).

**Table 2.** Profile of Respondents (n=311)

| Demographics | Frequency | Percentage (%) |
|---|---|---|
| **Gender** | | |
| Male | 191 | 61.4 |
| Female | 120 | 38.6 |
| **Age** | | |
| Under 18 years old | 27 | 8.7 |
| Age 19-30 | 61 | 19.6 |
| 31-45 years old | 87 | 27.9 |
| Age 45 and above | 136 | 43.8 |
| **Current Education Level** | | |
| Secondary school diploma | 20 | 6.4 |
| Associate Degree | 69 | 22.1 |
| Bachelor's degree | 131 | 42.1 |
| Graduate or above | 91 | 29.3 |
| **How many times have you been to the theater?** | | |
| 1-3 times | 36 | 11.6 |
| 4-6 times | 155 | 49.8 |
| 7-10 times | 88 | 28.3 |
| 10 and above | 32 | 10.3 |
| **Types of Programs** | | |
| Business | 60 | 19.3 |
| Engineering | 23 | 7.4 |
| Information Technology | 16 | 5.1 |
| Mass Communication | 80 | 25.7 |
| Pre-University | 69 | 22.2 |
| Others | 63 | 20.3 |
| **Total** | 311 | 100 |

Reviewer #1:
3.Unify Data Quantity: The actual number of questionnaire data is 311, but the article only mentions 300, which is inconsistent. Please verify the data, and if invalid questionnaires or abnormal data are excluded, clearly state the exclusion criteria and quantity to ensure the accuracy and reliability of the data.

Thank you very much for your thoughtful review. We apologize for the discrepancy in the number of questionnaire data mentioned in the article. Upon verification, we found that the actual number is 311. We have corrected this in the text and excluded invalid questionnaires based on predefined criteria (e.g., incomplete responses, apparent random answers). The exclusion criteria and the number of excluded questionnaires has been clearly stated to ensure data accuracy and reliability.

### 4.2 Participant Demographics

The participants of the questionnaire were audience members who had watched performances at two theaters in Guangzhou, and a total of 326 responses were received. After excluding 15 responses that did not meet the inclusion criteria, 311 valid responses were retained for analysis. This study used a random sampling method to investigate these audiences (the results are shown in Table 2).

Reviewer #1:
4.Standardize Data Files: The management of data files is not standardized, and the transparency is insufficient. It is suggested to upload the organized SPSS files and the. Sav files of the analysis process to open-source platforms such as Mendeley Data and fairsharing.org,and provide the database DOI number for easy access to the data by readers. The questionnaire appendix can be omitted, and the details can be integrated into the data link description.

Thank you for your insightful feedback. We acknowledge the need for standardized data management and transparency. We have uploaded the organized SPSS files and .sav files of the analysis process to Mendeley Data (Reserved DOI: 10.17632/x6hr8hyrr4.1]).The DOI number is provided for easy access by readers. The questionnaire appendix has been omitted, with relevant details integrated into the data link description.

Mendeley Data                                                     Find Research Data    My Data

## Your dataset is in moderation

After you submit a dataset for publication, our team of moderators check that everything is in order and if so they approve the dataset for publication. We aim to moderate all datasets within 2 business days from submission (excluding UK holidays). We'll be in touch to let you know when your dataset goes live.

Now increase the exposure of your data!

If you publish an article describing your dataset in Data in Brief, your research data will be peer reviewed and made more discoverable and easier to reuse by other researchers.

Data in Brief is an Open Access journal which welcomes papers describing data from all research areas.

Find out more

Reviewer #1:
5.Optimize Assumptions and Models: The logical relationships of the research assumptions are not clear, and the theoretical model does not deeply explore the mediating and moderating effects. It is not enough to simply attach significant coefficients to the assumptions. Please reorganize the assumptions to clarify the causal logic of the variables, such as refining the impact path of audience interaction on affective reactions.

We recognize the lack of clarity in the logical relationships of the research assumptions and the depth of the theoretical model. We have reorganized the assumptions to clarify the causal logic of the variables, refining the impact path of audience interaction on affective reactions. This reorganization enhances the theoretical foundation and clarifies the relationship between variables.

#### 4.5.3 Audience Interaction mediates the effect of affective reactions on Audience satisfaction

Model 1 in Table 9 ~~demonstrates the direct effect~~ shows the direct impact of affective reactions on audience ~~interaction, confirming that the affective reactions have a significant positive effect on audience interaction~~satisfaction, with a statistical significance coefficient of 0.142 * * (2.695), indicating a positive and significant relationship between the two. Model 2 introduces audience ~~satisfaction as a mediating variable and shows the significant effect of audience satisfaction on audience interaction, implying that audience satisfaction plays a role in the relationship between~~ expectations as an additional variable and reveals a significant impact on emotional feedback and audience satisfaction, with a coefficient of 0.296 * (6.404). In addition, audience satisfaction still holds significant importance, although the coefficient has slightly decreased to 0.108 (2.028). These findings suggest that affective reactions and audience ~~interaction, implying that audience satisfaction plays a mediating role in the relationship between affective reactions and~~ satisfaction play a crucial role in shaping the impact of audience interaction. In addition, Model 3 ~~further illustrates the interaction term between~~ is the most comprehensive among the three, including audience affective reactions and ~~audience satisfaction, which is significant, indicating a significant mediating role of audience satisfaction, i.e., the effect of affective reactions on audience interaction varies with different levels of AS. These results reveal the significant effects~~ satisfaction, as well as interaction. The

Reviewer #1:

6.In-depth Analysis and Discussion: The result analysis only simply describes the path coefficients and does not deeply interpret the reasons for the non-significant paths and the implications of the results. Please pay attention to the non-significant paths, such as analyzing the reasons for the lack of significant impact of lyrics content depth and artistry on affective reactions from the aspects of lyrics content depth and artistry;if possible, please construct a more comprehensive theoretical model,introducing mediating variables and moderating variables,such as cultural differences and individual differences, to better explain the phenomena and guide practice.

We appreciate the feedback on the result analysis and have conducted a more thorough interpretation. We have delved into the non-significant paths, particularly analysing the reasons for the lack of significant impact of lyrics content depth and artistry on affective reactions. We believe that the variables in this model are sufficient to support this study. In addition to mediating variables, we also have two moderating variables (such as scene design and audience interaction).

## 4.6 Discussion

~~Assuming path coefficients.~~

Through factor analysis, confirmatory analysis, moderation effect analysis, and path analysis of structural equation modeling, this study validated the hypothesis results and path coefficients shown in Table 11 and Figure 2. The study ~~revealed complex relationships between~~ unveiled intricate relationships among different variables,

Performer emotions have a significant positive impact on ~~emotional experience~~ affective reactions (H11), highlighting the importance of performer emotions in ~~stimulating audience emotional responses.~~ evoking emotional reactions from the audience.

The moderating effect of scene design on the relationship between performer's emotions and ~~emotional experience~~ affective reactions has been confirmed (H12)~~, indicating~~. This indicates that appropriate scene design can significantly enhance the communication of performer's emotions and the ~~emotional experience of the~~ audience's affective reactions. Regarding the non-significant paths, particularly the lack of significant impact of lyrics content depth and artistry on affective reactions, several potential reasons can be explored. It may be that the selected nursery rhymes, despite their cultural significance, did not resonate deeply with the audience in terms of emotional content or artistic expression. Additionally, the adaptation process may have altered the original essence of the rhymes in a way that weakened their emotional impact. Future research could delve deeper into these aspects, exploring how lyrics content depth and artistry can be better integrated into musical theater adaptations to evoke stronger affective reactions from the audience.

~~In summary~~Overall, this research ~~found that~~ underscores the importance of audience interaction and scene design ~~can enhance their emotional experience~~ in enhancing affective reactions and satisfaction. For future research, it is ~~particularly important to explore how to enhance~~ imperative to explore strategies for strengthening the connection between audiences and music ~~theatres~~ theaters through educational and cultural ~~intervention strategies, especially in~~ interventions, particularly in the context of adapt-

particularly ~~the key roles between~~ emphasizing the pivotal roles of audience interaction, ~~emotional experience~~affective reactions, audience satisfaction, and repurchase intention~~,~~ ~~including~~. The key findings include:

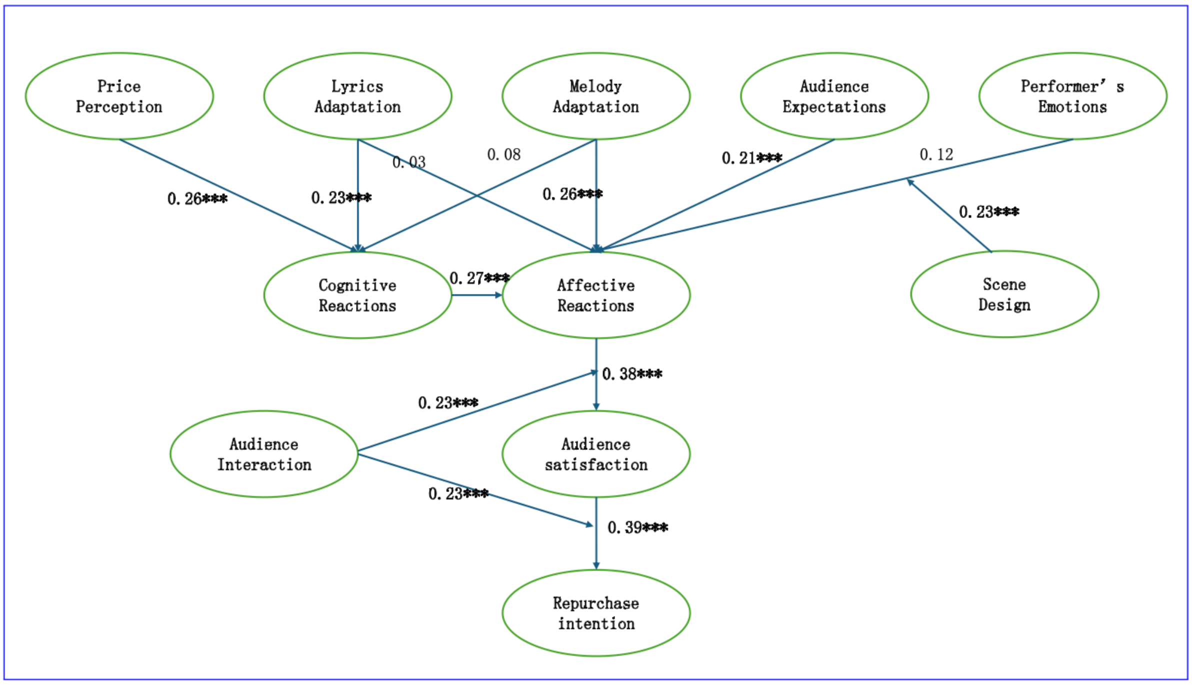


**Fig 2.** Assuming path coefficients.

There is a significant positive correlation (H1) between audience satisfaction and repurchase intention, ~~confirming the significant impact of audience satisfaction~~ reinforcing the notion that audience satisfaction has a substantial impact on repurchase behav-

Reviewer #1:

7.Improve Conclusion: The conclusion section is brief and does not fully summarize the research findings and contributions, and the guidance for future research directions is not clear. Please summarize the core findings in detail, such as the key role of audience interaction and emotional experience and their impact mechanisms on satisfaction and repurchase intention; point out the theoretical and practical significance of the research for the development of the musical theater industry, and propose future research questions based on the limitations, such as cross-cultural comparisons and long-term tracking of audience loyalty, etc.

We understand the need for a more comprehensive conclusion. We have expanded this section to summarize the core findings in detail, highlighting the key role of audience interaction and emotional experience and their impact mechanisms on satisfaction and repurchase intention. In addition to revising the summary, we also added a section (Limitations and Future Research) to the manuscript to enhance the readability of the article and lay the groundwork for future research.

# 7 Conclusion

This study empirically analyzed how musical adaptations of nursery rhymes influence audience interaction, ~~emotional experience~~affective reactions, satisfaction, and repurchase intentions. ~~Factor~~ Fac- tor analysis, validation analysis, and structural equation modeling identified audience interaction and ~~emotional experience~~ affective reactions as key contributors to satisfaction and repurchase intention. Specifically, the direct positive impact of audience interaction on affective reactions highlights the importance of engaging and immersive theatrical experiences. The results revealed that audience interaction directly enhanced ~~emotional experience~~affective reactions, which significantly boosted satisfaction and, consequently, repurchase intention. In the context of nursery rhyme adaptations, the emotional richness and cultural resonance of the content facilitated emotional connections, making audiences more likely to return. Although lyrical adaptation had no direct effect on ~~emotional experience~~affective reactions, it indirectly enhanced cognitive responses by improving price perception, thereby deepening the overall audience experience. Scenic design significantly enhanced performers ~~'~~ 'emotional expression and the audience's ~~emotional experience~~affective reactions, further increasing satisfaction and likelihood of returning. The theoretical significance of this study is that it helps to understand how musical adaptations effectively attract audiences through emotional and interactive elements. In fact, our research findings provide insights for musical producers and directors on how to create adaptations that resonate with audiences, cultivate loyalty, and repeat visits. Future research could provide additional avenues for investigating the role of other factors, such as social media engagement or word-of-mouth marketing, in promoting musical attendance. At the same time, it can provide a deeper understanding of the sustained impact of musical adaptations on audience engagement.

Reviewer #2:
1. The abstract effectively summarizes the study's objectives, methods, and findings. Consider emphasizing the practical implications for musical production more explicitly.

Thank you for your thorough and constructive review. We have revised the abstract to include a more prominent discussion on the practical implications. Specifically, we have added a sentence highlighting how our findings can inform strategies for enhancing audience engagement and satisfaction in musical theater productions, particularly those featuring adaptations of Chinese nursery rhymes.

## Abstract

Musicals, as an art form, are ~~gaining more attention due to the~~ attracting greater attention in China's increasingly competitive market~~in China~~. In particular, the adaptation of Chinese nursery rhymes in musicals and its influence on ~~the audience 's emotional experience~~ audience affective reactions and behavioral intentions has ~~become a significant research topic~~emerged as a notable research focus. This study aims to explore the impact of nursery rhyme adaptations in musicals on audience interaction, ~~emotional response~~affective reactions, satisfaction, and repurchase intention. ~~Furthermore, this study empirically analyzes~~ Using questionnaire surveys and structural equation modeling, we empirically analyse how audience interaction and ~~emotional experience in~~ affective reactions in these musical adaptations enhance satisfaction and increase repurchase intention~~, using questionnaire surveys and structural equation modeling~~. The results ~~show~~ indicate that audience interaction significantly ~~enhances emotional responses~~boosts affective reactions, leading to ~~improved satisfaction and increased repurchaseintention. Additionally~~heightened satisfaction and a greater likelihood of repurchase. Furthermore, elements such as scenic design, lyric adaptation, and melody adaptation ~~enhance~~ enrich the overall artistic experience by influencing both cognitive and ~~emotional~~ affective responses. This study ~~provides~~ offers strategic recommendations for musical production, ~~emphasizing~~ specifically highlighting the importance of audience interaction and ~~emotional responses~~ affective reactions throughout the creative process to maximize engagement and commercial success. In conclusion, ~~these findings offer~~ our findings provide valuable theoretical and practical insights that not only support cultural inheritance ~~and innovation~~ but also drive innovation within the musical theater industry.


Reviewer #2:

2. Introduction:

2.1 The introduction provides a strong rationale for the study but could benefit from a clearer articulation of how it fills specific gaps in the existing literature.

2.2 More context on the significance of nursery rhyme adaptations in Chinese musicals would strengthen the argument.

We have expanded the introduction to clearly delineate the specific gaps our study addresses. We now explicitly discuss how previous research has overlooked the impact of nursery rhyme adaptations in Chinese musicals on audience behavior and return rates, thereby situating our work within a distinct niche in the scholarly landscape. To provide deeper context, we have incorporated additional historical and cultural information on the role of nursery rhymes in Chinese society and their adaptation into musical theater. This augmentation underscores the importance of our research topic and its relevance to understanding contemporary audience preferences and behaviors.

# 1 Introduction

The diversification of products supplied by China's performing arts market and the growth of Chinese people's art consumption have made musicals a new growth point in the art market; hence, there is an urgent need to launch a number of new works that can cater to China's consumption demand for musicals, and to break down the linguistic barriers and competitive advantages of traditional musicals. Musicals now stand out in the competition in the performing arts market, and are expected to form an independent industrial chain, further enhancing the internal driving force of the cultural and creative industries and the performing arts market. Correspondingly, the musical industry experienced significant changes and impacts due to external factors such as the pandemic in 2022. For example, ~~Broadway's total box office revenue~~ Broadway's total box office revenue for the 2022-23 season was 1.578 billion, with an audience of 12.28 million and 1474 weeks of performance[1]~~. This data~~. This data has increased compared to the severely affected 2021-22 season by the epidemic, with box office revenue of 845 million and an audience of 6.73 million at that time[1]. In the West End of London, ~~the total box office revenue~~ the total box office revenue in 2022 was 892.8 million pounds, with a total of 19224 performances, an increase in revenue and number of performances compared to previous years[1]~~. These data highlight the gradual recovery trend~~. These data highlight the gradual recovery trend of the theater industry in response to external disturbances such as the pandemic and theater capacity restrictions. The report also shows that the average ticket price in the Western District has increased to 52.17 pounds[1]~~.~~

Nursery rhymes play multiple roles in Chinese society, including cultural inheritance, moral education, entertainment and leisure, and social reflection. In recent years, their adaptation in musicals has not only enriched the artistic form, but also injected new vitality into traditional culture. This adaptation reflects the contemporary audience's acceptance and aesthetic needs for traditional culture, and is of great significance for understanding audience preferences, promoting cultural inheritance, and stimulating artistic innovation. It is an important bridge connecting traditional culture and modern aesthetics and promoting the development of cultural undertakings.

~~Adapting teams should tap into the work's potential , create~~ The adaptation team should utilize the potential of these works, carefully craft unique scripts, ~~and~~ ensure high-quality ~~productions through~~ production through meticulous stage design, captivating music, and talented ~~acting, highlighting~~ performances, thereby emphasizing the artistic charm and cultural value of the original ~~. Therefore, musical adaptations are crucial, as they can enhance theater influence.~~ work.

~~Theoretically~~In theory, this study ~~offers new insights by showing that nursery rhyme adaptations in musicals promote audience repurchase intentions, an underexplored area~~ provides a new perspective by demonstrating that the adaptation of nursery rhymes in musicals promotes audience repurchase intention, which has received little attention in the literature (Muntean, 2020 [4]; Nijs et al., 2023 [5]). This ~~research also develops a detailed mechanism outlining~~ study further developed a comprehensive mechanism that outlines the interrelationships between various factors, with cognitive and affective reactions ~~as intermediaries and scenic design as a moderator~~acting as mediators and landscape design acting as moderators. Importantly, this study expands the scope of

Reviewer #2:

3. Methodology:

3.1 The methodology is detailed and appropriate. The explanation of questionnaire design and the use of validated scales ensure rigor. 3.2 It might be helpful to specify the demographic characteristics of the survey respondents to provide more context for the findings.

We appreciate the recognition of our methodological rigor and have no further changes to make in this section based on your feedback. We have added a new subsection within the methodology detailing the demographic characteristics of our survey respondents, including age, gender, education level, and frequency of attending musical theater performances. This information will help readers better understand the context in which our findings were obtained.

## 4.2 Participant Demographics

The participants of the questionnaire were audience members who had watched performances at two theaters in Guangzhou, and a total of 326 responses were received. After excluding 15 responses that did not meet the inclusion criteria, 311 valid responses were retained for analysis. This study used a random sampling method to investigate these audiences (the results are shown in Table 2).

**Table 2.** Profile of Respondents (n=311)

| Demographics | Frequency | Percentage (%) |
|---|---|---|
| **Gender** | | |
| Male | 191 | 61.4 |
| Female | 120 | 38.6 |
| **Age** | | |
| Under 18 years old | 27 | 8.7 |
| Age 19-30 | 61 | 19.6 |
| 31-45 years old | 87 | 27.9 |
| Age 45 and above | 136 | 43.8 |
| **Current Education Level** | | |
| Secondary school diploma | 20 | 6.4 |
| Associate Degree | 69 | 22.1 |
| Bachelor's degree | 131 | 42.1 |
| Graduate or above | 91 | 29.3 |
| **How many times have you been to the theater?** | | |
| 1-3 times | 36 | 11.6 |
| 4-6 times | 155 | 49.8 |
| 7-10 times | 88 | 28.3 |
| 10 and above | 32 | 10.3 |
| **Types of Programs** | | |
| Business | 60 | 19.3 |
| Engineering | 23 | 7.4 |
| Information Technology | 16 | 5.1 |
| Mass Communication | 80 | 25.7 |
| Pre-University | 69 | 22.2 |
| Others | 63 | 20.3 |
| **Total** | 311 | 100 |

Reviewer #2:
4. Results:
4.1 The results are well-presented, with clear tables and figures. However, the discussion of non-significant findings (e.g., the impact of lyrics adaptation on affective reactions) could be expanded to explore possible reasons.
4.2 The inclusion of a visual path model helps clarify the relationships among variables.

We have expanded the discussion of non-significant findings, particularly focusing on the impact of lyrics adaptation on affective reactions. We believe that the variables in this model are sufficient to support this study. In addition to mediating variables, we also have two moderating variables (such as scene design and audience interaction). Moreover， We have reorganized the research model diagram and presented it for better understanding.

## 7 Conclusion

This study empirically analyzed how musical adaptations of nursery rhymes influence audience interaction, ~~emotional experience~~affective reactions, satisfaction, and repurchase intentions. ~~Factor~~ Fac- tor analysis, validation analysis, and structural equation modeling identified audience interaction and ~~emotional experience~~ affective reactions as key contributors to satisfaction and repurchase intention. Specifically, the direct positive impact of audience interaction on affective reactions highlights the importance of engaging and immersive theatrical experiences. The results revealed that audience interaction directly enhanced ~~emotional experience~~affective reactions, which significantly boosted satisfaction and, consequently, repurchase intention. In the context of nursery rhyme adaptations, the emotional richness and cultural resonance of the content facilitated emotional connections, making audiences more likely to return. Although lyrical adaptation had no direct effect on ~~emotional experience~~affective reactions, it indirectly enhanced cognitive responses by improving price perception, thereby deepening the overall audience experience. Scenic design significantly enhanced performers ~~’~~‘emotional expression and the audience’s ~~emotional experience~~affective reactions, further increasing satisfaction and likelihood of returning. The theoretical significance of this study is that it helps to understand how musical adaptations effectively attract audiences through emotional and interactive elements. In fact, our research findings provide insights for musical producers and directors on how to create adaptations that resonate with audiences, cultivate loyalty, and repeat visits. Future research could provide additional avenues for investigating the role of other factors, such as social media engagement or word-of-mouth marketing, in promoting musical attendance. At the same time, it can provide a deeper understanding of the sustained impact of musical adaptations on audience engagement.

### 3.11 Conceptual model

The conceptual model is shown as Figure 1.

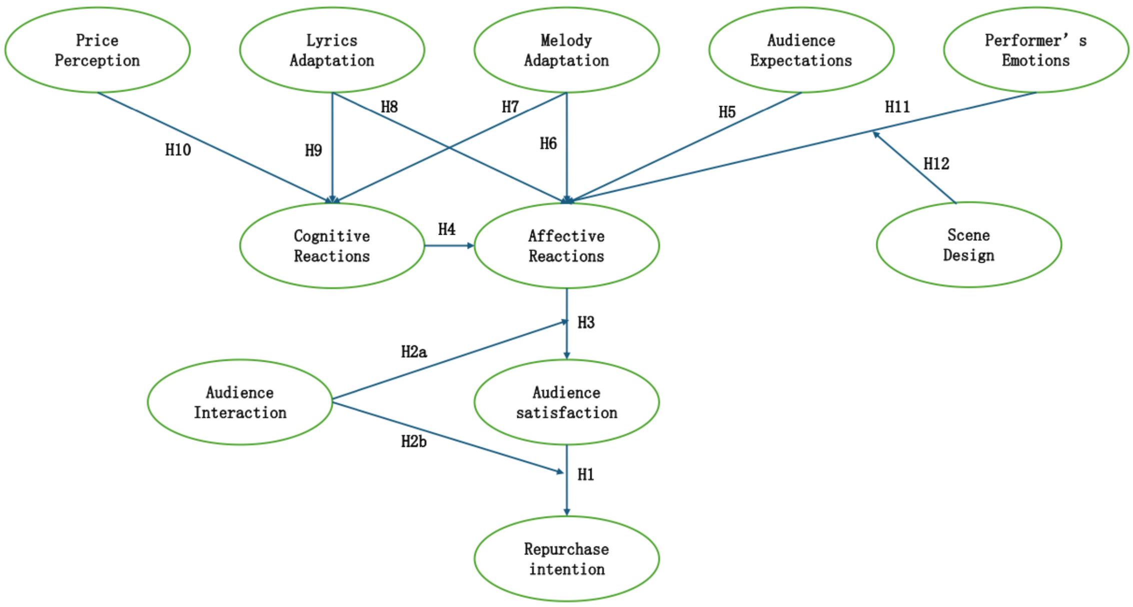


**Fig 1.** Research model.

Reviewer #2:

5. Discussion:

5.1 The discussion effectively interprets the findings but could be improved by comparing the results more extensively with those of prior studies.

5.2 Consider addressing limitations such as the generalizability of findings beyond the surveyed audience.

Thank you for your thorough review and valuable suggestions. We have incorporated a more extensive comparison of our results with those from prior studies, discussing both similarities and differences. This expanded discussion contextualizes our findings within the broader scholarly conversation and highlights the novel contributions of our work. Moreover, we have added a new paragraph in the discussion addressing potential limitations, particularly focusing on the generalizability of our findings. We acknowledge that our sample may not be representative of all musical theater audiences and suggest directions for future research to explore the applicability of our findings in different demographic and cultural contexts.

# 6 Limitations and Future Research

There are some important caveats to the research that deserve attention. First, the samples used in this study were targeted at the surveyed audience, which may limit the applicability of the results to a wider population. Future research could benefit from expanding the geographical and demographic scope of the sample to validate the research findings and ensure their robustness across different audiences. Next, the study also focused on specific elements such as landscape design, lyrics adaptation, and melody adaptation. Although these factors are crucial, there may be other factors that affect audience engagement and satisfaction in musical adaptations, which were not explored in this study. Future research can delve deeper into other potential variables, such as director selection, actor performance, or cultural background, to gain a more comprehensive understanding of the driving factors behind audience responses. Future research can also incorporate cross-cultural comparisons, rather than being limited to one's own culture to promote cultural exchange between countries. Third, the methods used in this study rely on questionnaire surveys and structural equation modeling. Although these methods are highly suitable for exploring the relationships between variables of interest, they do not provide in-depth qualitative insights into the audience's experience and perception. Combining qualitative research methods such as interviews or focus groups can provide richer data and a deeper understanding of audience reactions to musical adaptations. Finally, future research should address these limitations by examining a wider and more diverse sample, considering other influencing factors, and combining qualitative research methods to gain a more comprehensive understanding of audience reactions in the musical industry.

Reviewer #2:

6. Practical Implications:

6.1 The recommendations for enhancing audience interaction and satisfaction are practical and grounded in the findings.

6.2 Expanding on how these insights could be applied to different cultural or economic contexts would add value.

We appreciate the recognition of the practicality of our recommendations. Meanwhile, we have expanded our discussion on practical implications to include specific examples of how our findings could be applied in various cultural and economic settings. This includes considerations for different marketing strategies, audience engagement tactics, and potential collaborations with local artists or cultural institutions to tailor adaptations to specific regional contexts.

## 5.2 Practical implications

The practical strategy of adapting Chinese nursery rhymes into musicals emphasizes the importance of considering the diverse backgrounds, cultural preferences, and viewing styles of the audience. The production team can enhance audience engagement and satisfaction by designing interactive activities that align with their cultural and ~~emotional experiences~~affective reactions. This is not only applicable to large theaters, but also to audiences with different economic conditions, ensuring that everyone can enjoy the charm of musicals. Although high-end stage equipment can provide abundant performance resources, the improvement of audience satisfaction depends more on personal investment, cultural literacy, and production quality. In addition, according to (Wu(2022) [62]), effective ~~audience~~ au- dience interaction strategies significantly enhance emotional engagement and satisfaction; Tronstad, Tronstad(2022) pointed out that modern adaptation can attract more young audiences; Omar [63], Wang et al.(2023) emphasizes that high-quality visual and auditory elements can enhance the audience experience [64].

Moreover, in areas with rich cultural heritage, incorporating local cultural elements into music adaptations by staff may resonate more deeply with the audience. Meanwhile, innovative solutions such as community-based production or digital platforms can be explored to make musicals more accessible in economies with limited resources. Based on these studies, we provide a series of evidence-based practical recommendations to promote the development of the musical industry and optimize the audience experience.
